# COVID-19 Lessons From The Field: Toward A Pediatric Physical Therapy Telehealth Framework

**DOI:** 10.5195/ijt.2022.6448

**Published:** 2022-06-03

**Authors:** Galia Daube Fishman, Jeananne Elkins

**Affiliations:** 1 Department of Physical Therapy, Movement & Rehabilitation Sciences, Northeastern University, Boston, Massachusetts, USA; 2 University of Haifa, Mount Carmel, Haifa, Israel

**Keywords:** Early intervention, Pediatric physical therapy, Telehealth

## Abstract

**Introduction:**

Telehealth is an established health service delivery method, yet little is known regarding pediatric physical therapy telehealth. We aimed to evaluate users' experiences and create a framework for effective delivery.

**Methods:**

A pediatric physical therapists' telehealth user survey was conducted.

**Results:**

Seventy-three respondents varied in years of experience and caseload. Most found telehealth easy to learn and use, and they believed the treatment they delivered was useful. Three main themes for successful treatments emerged and were organized into a framework for effective delivery: (1) caregivers' involvement; (2) therapist telehealth ‘toolbox’ (sub-divided into: treatment management tools, and therapist-caregivers' collaboration tools); and (3) telehealth client (child) characteristics.

**Conclusion:**

This study suggests a new framework for effective pediatric physical therapy telehealth delivery to support best practice, for use by administrators and therapists, and recommends directions for further research.

By using electronic information and telecommunications technologies, telehealth can support and promote health care for patients (American Physical Therapy Association, 2019; Cady et al., 2015; Lee & Harada, 2012; U.S. Department of Health and Human Services, 2020). While historically telehealth has served a niche market with limited coverage by insurance companies, telehealth emerged as a solution during the COVID-19 pandemic “stay-at-home” orders. The temporary approval to use and bill Medicare and Medicaid for real-time services (American Physical Therapy Association, 2020; U.S. Department of Health and Human Services, 2020) eliminated many of the previous barriers for pediatric physical therapists (PTs) (Long et al., 2015).

While not used widely in pediatric physical therapy, telehealth interventions have been successful in both pediatric speech therapy and occupational therapy (Behl et al., 2017; Cason et al., 2018; Criss, 2013; Figueiredo, 2019; Meadan & Daczewitz, 2015). Overall, in pediatric speech therapy and occupational therapy, internet-based treatment is clinically effective and results in high family participation and satisfaction as well as being cost-effective (Behl et al., 2017; Hageman, 2020; Lee & Billings, 2016; Lee & Harada, 2012). The telehealth model for school-based therapy, outpatient therapy, and early intervention fits both the daily lives of the families (Cole et al., 2019) and the home equipment available (Tenforde et al., 2020). Telehealth has been found to support and empower coaching behaviors of the providers for family-centered early intervention (Stredler-Brown, 2017), and supports daily routines focusing on therapy. Telehealth has also been found to improve clinical flexibility and decrease cancellations (Cole et al., 2019; Zylstra, 2013).

Yet, opposition to pediatric telehealth is not uncommon among PTs and families. Some providers describe limited efficacy and negative family attitudes about telehealth. Technical complaints are common (Cole et al., 2019).

Stronger evidence for pediatric physical therapy telehealth is essential. This research seeks to understand facilitators, barriers, and challenges in pediatric telehealth. Moreover, we seek to establish a framework for the telehealth delivery of effective pediatric physical therapy.

## METHODS

After receiving Northeastern University IRB# CPS20-08-11 approval, we posted an invitation on social media platforms for pediatric PTs to participate in the survey. Data were collected in September and October 2020. The online survey consisted of both quantitative and qualitative sections. In the quantitative section, the participants answered demographic questions and yes/no questions for their agreement or disagreement with 13 statements regarding their telehealth work conditions, opinions, and feelings. The four-part qualitative section consisted of three open-ended questions: (1) “What tips or advice would you like to share with other PTs about teletherapy?” (2) “Can you describe your ‘ideal’ telehealth patient and family?” (3) “Thinking about your patients who have best adjusted to telehealth, why are these sessions most effective?” and (4) a space to provide any other information the PT would like to offer.

We used descriptive statistics to describe the sample from the quantitative data. A thematic analysis was conducted from the qualitative data. Both researchers independently coded the responses. When there was a disagreement the researchers met to discuss and agree upon a code. The codes were then organized into themes. From the themes, a framework for effective delivery of pediatric physical therapy telehealth was constructed.

## RESULTS

### SAMPLE DESCRIPTIVE

We received and included seventy-three (N=73) responses from pediatric PTs with a range of work experience from one to 48 years. As shown in Table 1, the respondents reported an average of nine weekly telehealth appointments. On average, the PTs communicated with their clients more than two times between sessions.

**Table 1:** Descriptive Statistics (n=73)

Variable	Mean	Std. Dev.	Range
Weekly telehealth appointments	9.6	8.19	1-40
Number of communications between appointments	2.3	2.0	0-10
Years of work	15.7	12.1	1-48

As shown in Table 2 the majority of PTs (89%) found the online platforms easy to learn and use. Further, most PTs reported having enough space to deliver telehealth (68%) as well as having the right telehealth equipment (69%). Only one-fourth of the respondents (26%) reported more cancellations than during in-person delivery. About a quarter of respondents (26%) believed that teletherapy was not an effective method for providing pediatric physical therapy though most felt that the treatment they delivered was useful (79%). More than half of the respondents (57%) reported that they were seeking out and trying new equipment and new activities for their teletherapy sessions, and 59% reported that they sent their clients' families videos, handouts, or activities before and after live telehealth sessions. Families/caregivers were present when needed for most of the therapy sessions (87%). Of interest, only 38% of respondents answered that they enjoy providing physical therapy through telehealth.

**Table 2:** Telehealth Questions (n=73)

	Number	Frequency (%)
I have more cancellations in telehealth than in in-person	19	26%
The telehealth I'm delivering is useful for my clients.	58	79%
The platform I use for telehealth was easy to learn and use.	65	89%
I enjoy providing physical therapy through telehealth.	28	38%
I am seeking out and trying new equipment and new activities for my telehealth meetings.	42	57%
I have the right equipment to use in my telehealth sessions (good camera, earphones, computer, etc.)	51	69%
Before and after the live telehealth visit, I send the patient's family handouts, videos, or activities.	43	59%
In general, I feel that telehealth is not an effective method for providing pediatric physical therapy.	19	26%
In most of my telehealth meetings, the family/caregivers are present when needed.	64	87%
I have enough space at my clinic/home for telehealth sessions.	50	68%
I'm the owner of the practice.	9	12%

### QUALITATIVE ANALYSIS

In the open-ended survey's responses, three main themes about effective and successful telehealth delivery in pediatric physical therapy emerged from the data. The themes were: (1) caregiver's involvement, (2) the telehealth toolbox for the physical therapist, and (3) client (child's) characteristics.

#### THEME 1: CAREGIVER’S INVOLVEMENT

According to the respondents, a caregiver's active participation is the primary factor for a successful and effective pediatric physical therapy telehealth treatment. The caregiver is essential for setting up the equipment as well as establishing the network connection.

As one of the respondents explained:

“The most important factor is parent involvement. If they are not willing to participate and assist the child, it will be unsuccessful.”

Essential caregiver behaviors during the telehealth treatment are being actively present, asking questions, and exhibiting a motivation to learn. One respondent with 17 years of practice described the importance of a family's support:

“Family will be involved and try all activities at least once on screen…family support is essential for a successful session.”

Another described successful sessions when parents:

“Engage in brainstorming and implementation of intervention strategies.”

Supportive family behavior enabled a clinician's transition to telehealth:

“An involved family that is willing to be supportive as I'm learning teletherapy.”

Setting up the virtual session, including having a quiet and safe area for the session, was mentioned by many of the survey's respondents. An experienced PT (i.e., 21-years of practice in early intervention) suggested to new therapists in telehealth:

“I have the parent set up area ahead of time, blanket on floor, camera set to see wide view, a few toys handy.”

Survey responders reported that communicating between appointments, reading messages, and implementing a home exercise program were behaviors that supported successful transition to telehealth physical therapy. One PT described effective parental communication:

“These parents have embraced sessions by video. They have communicated with videos between sessions to set up what they needed help with. They have embraced suggestions, handouts, videos that I have given them, and they have implemented those suggestions in the daily activities.”

#### THEME 2: PHYSICAL THERAPY TELEHEALTH TOOLBOX

**Telehealth management tools.** Flexibility and patience were the two most important management tools for physical therapists. As well, to: “…try new things!”

Planning the sessions in advance was also important. As one respondent explained:

“Set-up is key to success.”

“Be flexible and prepared with lots of different activities.”

Learning multiple software options and new activities (e.g., digital and physical games for online engagement) was highly recommended. One respondent suggested:

“Be over-prepared with activities that you can do that day (more than in-person visits). I find that kids lose interest faster in activities when done via teletherapy than they do with in-person visits.”

**Therapist-caregiver collaboration tools.** One respondent stated:

“The parents are my hands.”

Proactive communication efforts can strengthen the therapist-caregivers partnership. These include open and continuous communication both during and between the virtual appointments. Communication should include session plans before the visit and sending reminders and ideas for daily routines and daily practice:

“Re-confirm every single appointment”

“Plan your sessions and share with parents what you need prior to the session.”

Also noted, was the need to communicate the appropriate amount of information:

“Always meet the family where they are- many are overwhelmed, don't overwhelm them more.”

The respondents recommended encouraging the caregiver to ask questions as well as supporting caregivers with handling techniques, positioning, active practice, and play. They recommended practical demonstrations using videos and a doll. A physical therapist with 40 years of practice stated:

“An ideal session is one that makes me feel that by whatever I said or instructed them to do with their child/baby that they had that “ah ha” moment and really understood what they needed to do to improve their child's motor abilities. Additionally, to really be an ideal session, I would also like to see the child responding to their parent's handling and facilitation in a way that improves the quality of their movement. It is icing on the cake when the child/baby works hard and is happy doing it.”

We learned from the respondents that the use of coaching techniques in their prior in-person practice helped both families and therapists to transition to telehealth:

“Learn to use the coaching model – it lends itself very well to teletherapy and the kids have great results “

“Coaching is helpful when providing teletherapy. It allows you to really engage families in rehab care”

A response from an early intervention PT emphasized the coaching concepts in her telehealth sessions:

“Ask the families what would be helpful to work on this week. If it's important to them, try to think of lots of ideas for activities for them to do with the child and siblings since they may have more time at home. In early intervention don't worry too much about entertaining the child, but really work on coaching the family.”

#### THEME 3: PATIENT-CHILD’S CHARACTERISTICS

The patient's (the child) characteristics were almost never mentioned by our respondents, as an important aspect of successful telehealth treatment. A few participants stated the best age range to fit the pediatric telehealth practice model is babies and older children who can follow directions. One PT stated her ideal patient is:

“Either an infant who isn't moving a lot or an older (5 years or above) child who can follow directions and families who are eager to learn and work with their child.”

### FRAMEWORK FOR SUCCESSFUL PEDIATRIC TELEHEALTH

**Figure 1:**
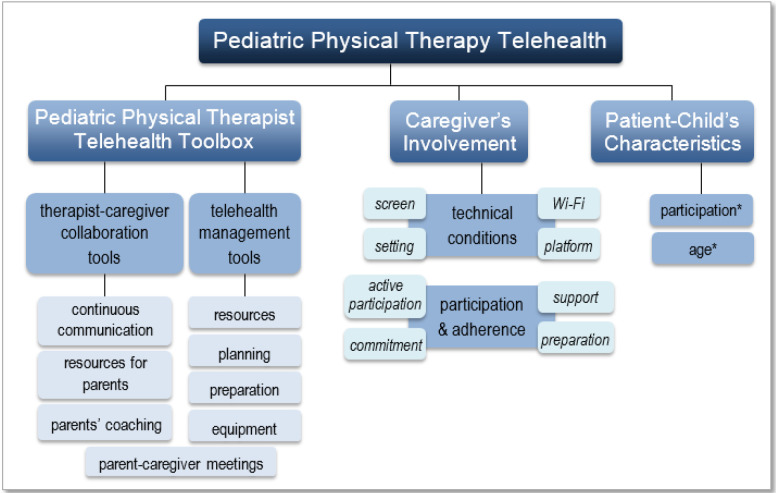
Pediatric Physical Therapy Telehealth Framework for Effective Delivery

As shown in Figure 1, we developed a framework for successful pediatric physical therapy telehealth. This framework is based on the recommendations of the American Physical Therapy Association for telehealth (American Physical Therapy Association, 2019) as well as our survey's qualitative analysis, and published literature. This framework can assist in the preparation, education, and support of best practices in pediatric physical therapy telehealth delivery.

*For continued future evaluation

## DISCUSSION

Pediatric PTs reported the available telehealth platforms easy to use. Space for the treatment in the clients' home was not a problem. Most participants believed their telehealth delivery was useful to their clients; moreover, the PTs put effort into this new method of treatment. The PTs reported working hard to learn new virtual activities, to communicate with the families, and to provide resources between the scheduled treatments. Similar results were found in another recent physical therapy survey, in which respondents described putting much of their time into planning or preparation of activities and accessing telehealth resources (Hall et al., 2021).

Inadequate equipment in the home was identified as a barrier to telehealth treatment (Meadan & Daczewitz, 2015). PTs reported difficulties when a caregiver used a smartphone instead of a tablet or computer. The use of smartphones presented two types of challenges. First, it can be difficult for the child and/or caregiver to view the therapist's demonstration of the treatment or skills. Second, the PT may have difficulty analyzing the child's activities and movements. Schools and clinics should therefore consider loaning the parent or caregiver a computer to use for access to the telehealth treatments (Lee & Billings, 2016; Reifenberg et al., 2017).

While the remote practice of physical therapy presents challenges that include minimal control over the child's environment and cooperation levels, such challenges can be overcome with appropriate strategies. Flexibility in the treatment session as well as having alternatives to the planned session activities are essential when a session does not go as initially planned.

Strengthening the caregiver's role and participation through enhanced communication between the therapist and the caregiver can improve the child's behavior and participation.

Telehealth interaction requires the PT to develop a new set of communication skills (Lee & Billings, 2016). Optimizing collaboration with childs' caregivers was one of the main themes to ensure successful telehealth delivery. Many PTs recommended using the family-centered and coaching model that is employed in early intervention services; these supported a successful transition to telehealth from in-person service. The coaching method used in early intervention is an expert-based approach emphasizing meaningful relationships and collaboration between the coach and the child's caregiver. The coaching method is goal-oriented and based on adult learning principles (Friedman et al., 2012; Rush, 2019). This “triad intervention” focuses on strengthening parent-child relationships. Virtual visits created a good setting for effective use of parent coaching, and teletherapy for young children was reported to be more flexible in the scheduling of sessions (Cole et al., 2019; Hageman, 2020). Teletherapy enabled home activities and daily routines practiced in the child's and family's environment in real-time (Meadan & Daczewitz, 2015).

While we expected the child's characteristics to be of primary importance in the telehealth treatment, our survey participants did not identify this as a primary concern. The fact that this survey took place during a global pandemic might explain why the PTs rarely mentioned it. Our respondents were coping, adapting, and tailoring a plan of care to each and every child to keep the therapy routine on track. Although some respondents shared a recommendation to use telehealth only with children who can follow directions or for older children, some successful telehealth studies regarding communication and behavioral interventions may suggest that using our Therapist-Caregivers' Collaboration Tools can support telehealth physical therapy delivery for clients of elementary school age and younger (Monlux et al., 2019; Tsami et al., 2019). Most answers referenced the family's involvement more than the child's compliance. Future research might examine each childs' cooperation, active participation, and adherence as well as detail their diagnosis, age, and level of function.

We found an interesting result regarding enjoyment of the telehealth service delivery model. Most PTs surveyed (79%) reported the teletherapy they delivered was useful to their clients, a finding that is consistent with other physical therapy telehealth research conducted during the COVID-19 pandemic (Hall et al., 2021; Tenforde et al., 2020). However, only 38% of our respondents reported enjoyment in delivering PT services through telehealth. Job satisfaction is an important part of evaluating telehealth practice (Garcia & Adelakun, 2019), and many reasons may affect this finding. Satisfaction rate may relate to the COVID-19 pandemic and its impact on the PTs emotional states. We hope that implementing our new pediatric physical therapy telehealth framework will promote effective delivery and will improve job satisfaction.

## CONCLUSION

This study sought to learn from users' experiences in pediatric physical therapy telehealth. We suggest a new framework for effective pediatric physical therapy telehealth delivery. The Pediatric Physical Therapy Telehealth Framework can support best practices, help the physical therapists deliver effective telehealth sessions, and evaluate client applicability for telehealth.

Further research is needed to better understand the client attributes needed for effective telehealth as well as the driving forces for PT satisfaction with telehealth.

## References

[B1] American Physical Therapy Association. (2019). Position on Telehealth. https://www.apta.org/apta-and-you/leadership-and-governance/policies/telehealth

[B2] American Physical Therapy Association. (2020). Telehealth state and federal regulations and legislation. https://www.apta.org/your-practice/practice-models-and-settings/telehealth-practice/state-regulations Updated September 28, 2020. Accessed November 22, 2020. (2020, September 28). APTA. https://www.apta.org/your-practice/practice-models-and-settings/telehealth-practice/state-regulations

[B3] Behl, D.D., Blaiser, K., Cook, G., Barrett, T., Callow-Heusser, C., Brooks, B.M., Dawson, P., Quigley, S., & White, K. R. (2017). A multisite study evaluating the benefits of early intervention via telepractice. *Infants and Young Children*, 30(2), 147–161. 10.1097/iyc.0000000000000090

[B4] Cady, R.G., Cady, R.G., Erickson, M., Erickson, M., Lunos, S., Lunos, S., Finkelstein, S.M., Finkelstein, S.M., Looman, W., Looman, W., Celebreeze, M., Celebreeze, M., Garwick, A., & Garwick, A. (2015). Meeting the needs of children with medical complexity using a telehealth advanced practice registered nurse care coordination model. *Maternal and Child Health Journal*, 19(7), 1497–1506. 10.1007/s10995-014-1654-1 25424455 PMC4480777

[B5] Cason, J., Hartmann, K., Jacobs, K., & Richmond, T. (2018). Telehealth in occupational therapy. *The American Journal of Occupational Therapy*, 72(Supplement_2), 7212410059p1-7212410059p18. 10.5014/ajot.2018.72S219 30674404

[B6] Cole, B., Pickard, K., & Stredler-Brown, A. (2019). Report on the use of telehealth in early intervention in Colorado: Strengths and challenges with telehealth as a service delivery method. *International Journal of Telerehabilitation*, 11(1), 33–40. 10.5195/ijt.2019.6273 31341545 PMC6597149

[B7] Criss, M. J. (2013). School-based telerehabilitation in occupational therapy: Using telerehabilitation technologies to promote improvements in student performance. *International Journal of Telerehabilitation*, 5(1), 39–46. 10.5195/ijt.2013.6115 25945212 PMC4296837

[B8] Figueiredo, M. (2019). The use of telehealth in pediatric occupational therapy. *Annals of Medicine (Helsinki)*, 51(sup1), 66–66. 10.1080/07853890.2018.1561616

[B9] Friedman, M., Woods, J., & Salisbury, C. (2012). Caregiver coaching strategies for early intervention providers: Moving toward operational definitions. *Infants and Young Children*, 25(1), 62–82. 10.1097/IYC.0b013e31823d8f12

[B10] Garcia, R., & Adelakun, O. (2019). A conceptual framework and pilot study for examining telemedicine satisfaction research. *Journal of Medical Systems*, 43(3), 51. 10.1007/s10916-019-1161-4 30684065

[B11] Hageman, J. (2020). The emergence of pediatric telehealth as a result of the COVID-19 pandemic. *Pediatric Annals*, 49, e283–e284. 10.3928/19382359-20200626-01 32674163

[B12] Hall, J.B., Luechtefeld, J.T., & Woods, M. L. (2021). Adoption of telehealth by pediatric physical therapists during COVID-19: A survey study. *Pediatric Physical Therapy*, 33(4), 237–244. 10.1097/PEP.0000000000000817 34323864 PMC8478094

[B13] Jason T Long, Christopher J Kovacs, Aurora J Hoobler, Erin E K Fritts, Brian E Cunningham, & Cailee M Caldwell. (2015). Integrating telehealth: Experiences in incorporating telehealth tools and principles into a pediatric therapeutic environment. *OT Practice*, 20(7), CE1–.

[B14] Lee, A.C.W., & Billings, M. (2016). Telehealth implementation in a skilled nursing facility: Case report for physical therapist practice in Washington. *Physical Therapy*, 96(2), 252–259. 10.2522/ptj.20150079 26658151

[B15] Lee, A.C.W., & Harada, N. (2012). Telehealth as a means of health care delivery for physical therapist practice. *Physical Therapy*, 92(3), 463–468. 10.2522/ptj.20110100 22135703

[B16] Meadan, H., & Daczewitz, M. E. (2015). Internet-based intervention training for parents of young children with disabilities: A promising service-delivery model. *Early Child Development and Care*, 185(1), 155–169. 10.1080/03004430.2014.908866

[B17] Monlux, K.D., Pollard, J.S., Bujanda Rodriguez, A. Y., & Hall, S. S. (2019). Telehealth delivery of function-based behavioral treatment for problem behaviors exhibited by boys with Fragile X Syndrome. *Journal of Autism and Developmental Disorders*, 49(6), 2461–2475. 10.1007/s10803-019-03963-9 30937736

[B18] Reifenberg, G., Gabrosek, G., Tanner, K., Harpster, K., Proffitt, R., & Persch, A. (2017). Feasibility of pediatric game-based neurorehabilitation using telehealth technologies: A case report. *The American Journal of Occupational Therapy*, 71(3), 7103190040p1–7103190040p8. 10.5014/ajot.2017.024976 28422630

[B19] Rush, D. D. (2019). The Early Childhood Coaching Handbook (Second Edition.). Paul H. Brookes Publishing Co. https://ebookcentral.proquest.com/lib/northeastern-ebooks/detail.action?docID=5942992

[B20] Stredler-Brown, A. (2017). Examination of coaching behaviors used by providers when delivering early intervention via telehealth to families of children who are deaf or hard of hearing. *Perspectives of the ASHA Special Interest Groups*, 2(9), 25–42. 10.1044/persp2.SIG9.25

[B21] Tenforde, A.S., Borgstrom, H., Polich, G., Steere, H., Davis, I.S., Cotton, K., O'Donnell, M., & Silver, J. K. (2020). Outpatient physical, occupational, and speech therapy synchronous telemedicine: A survey study of patient satisfaction with virtual visits during the COVID-19 pandemic. *American Journal of Physical Medicine & Rehabilitation*, 99(11), 977–981. 10.1097/PHM.0000000000001571 32804713 PMC7526401

[B22] Tsami, L., Lerman, D., & Toper-Korkmaz, O. (2019). Effectiveness and acceptability of parent training via telehealth among families around the world. *Journal of Applied Behavior Analysis*, 52(4), 1113–1129. 10.1002/jaba.645 31565804

[B23] U.S. Department of Health and Human Services. (2020, March 20). OCR Issues Guidance on Telehealth Remote Communications Following Its Notification of Enforcement Discretion. HHS.Gov. https://public3.pagefreezer.com/content/HHS.gov/31-12-2020T08:51/https://www.hhs.gov/about/news/2020/03/20/ocr-issues-guidance-on-telehealth-remote-communications-following-its-notification-of-enforcement-discretion.html

[B24] Zylstra, S. E. (2013). Evidence for the use of telehealth in pediatric occupational therapy. *Journal of Occupational Therapy, Schools & Early Intervention*, 6(4), 326–355. 10.1080/19411243.2013.860765

